# Experimental and Clinical Treatment of Chagas Disease: A Review

**DOI:** 10.4269/ajtmh.16-0761

**Published:** 2017-10-02

**Authors:** Policarpo Ademar Sales Junior, Israel Molina, Silvane Maria Fonseca Murta, Adrián Sánchez-Montalvá, Fernando Salvador, Rodrigo Corrêa-Oliveira, Cláudia Martins Carneiro

**Affiliations:** 1 Centro de Pesquisas René Rachou, FIOCRUZ, Belo Horizonte, Minas Gerais, Brazil;; 2 Infectious Diseases Department, Vall d’Hebron University Hospital, Universitat Autònoma de Barcelona, PROSICS Barcelona, Barcelona, Spain;; 3 Laboratório de Imunopatologia, Núcleo de Pesquisas em Ciências Biológicas, Universidade Federal de Ouro Preto, Ouro Preto, Minas Gerais, Brazil

## Abstract

Chagas disease (CD) is caused by the protozoan parasite *Trypanosoma cruzi* that infects a broad range of triatomines and mammalian species, including man. It afflicts 8 million people in Latin America, and its incidence is increasing in nonendemic countries owing to rising international immigration and nonvectorial transmission routes such as blood donation. Since the 1960s, the only drugs available for the clinical treatment of this infection have been benznidazole (BZ) and nifurtimox (NFX). Treatment with these trypanocidal drugs is recommended in both the acute and chronic phases of CD. These drugs have low cure rates mainly during the chronic phase, in addition both drugs present side effects that may result in the interruption of the treatment. Thus, more efficient and better-tolerated new drugs or pharmaceutical formulations containing BZ or NFX are urgently needed. Here, we review the drugs currently used for CD chemotherapy, ongoing clinical assays, and most-promising new experimental drugs. In addition, the mechanism of action of the commercially available drugs, NFX and BZ, the biodistribution of the latter, and the potential for novel formulations of BZ based on nanotechnology are discussed. Taken together, the literature emphasizes the urgent need for new therapies for acute and chronic CD.

## INTRODUCTION

Chagas disease (CD) or American trypanosomiasis is a potentially life-threatening zoonosis with the flagellate protozoan *Trypanosoma cruzi* as its etiological agent. An estimated eight million people in Latin America are infected with this parasite,^1^ and 100 million living in endemic areas (i.e., 25% of the total population in Latin America) are at risk of infection.^2^ Human infection also occurs in nonendemic areas because of growing international immigration and nonvectorial transmission routes such as blood transfusion, organ transplantation, and congenital infection.^3^ In addition, orally transmitted CD has been detected in endemic areas such as the Brazilian Amazon because of food carrying *T. cruzi* that originated from sylvatic triatomines.^4^

*Trypanosoma cruzi* has four developmental stages: the replicative epimastigote and amastigote stages, and the infective nonreplicative metacyclic and bloodstream trypomastigote stages. Infection begins when metacyclic trypomastigotes in the excreta of reduviid insects penetrate the bite wound; after entering the host cell, they transform into amastigotes that after several cycles of binary division in the cytoplasm differentiate into bloodstream trypomastigotes.^5^ When released from the host cell on rupture of the cell membrane, the bloodstream trypomastigotes infect neighboring cells and, owing to their dissemination throughout the blood, cells at other locations in the body. Amastigotes can also infect cells. Innate and acquired immune responses are critical for the control of *T. cruzi* and involve macrophages, natural killer cells, natural killer T cells, T and B lymphocytes, and the production of pro-inflammatory Th-1 cytokines such as interferon-γ (IFN-γ), tumor necrosis-α, and interleukin-12.^6^

CD has two clinical phases. The short acute phase is mainly oligosymptomatic but sometimes involves flulike symptoms and is defined by patent parasitemia. The chronic phase is characterized by fluctuating parasitemia, although most patients remain asymptomatic after several months and even decades, characterizing the indeterminate form of CD. Approximately 30–40% develop clinical symptoms characteristic of this phase, with the majority experiencing different levels of cardiac and/or digestive tract pathologies^7,8^ (cardiac and digestive forms of CD), which might be attributable to autoimmunity initiated by molecular mimetization.^9^

A drawback of the studies assessing the efficacy of BZ in the chronic phase of CD is the lack of a marker to define cure. Current recommendations rely on the switch of serology from positive to negative; however, this may take many years, precluding its use in clinical trials. Detection of *T. cruzi* DNA in peripheral blood allows having a rapid result, but it cannot be used to define cure. *Trypanosoma cruzi* DNA only serves as a tool to identify treatment failure because a negative result does not mean absence of infection. Moreover, long prospective studies to assess the value of a persistent negative polymerase chain reaction (PCR) after treatment are lacking.

CD treatment has been explored using two approaches: development of a preventive vaccine and identification of new effective drugs. Currently, no vaccines for CD are available or undergoing clinical tests. The present treatment of CD, used for > 40 years, is based on the nitroheterocyclic compounds nifurtimox (NFX; 3-methyl-4-[5′-nitrofurfurylideneamine]tetrahydro-4H-1,4-tiazine-1,1-dioxide; Bayer 2502; Bayer, Leverkusen, Germany) and benznidazole (BZ; N-benzyl-2-nitroimidazole acetamide; RO7-1051; Laboratório Farmacêutico do Estado de Pernambuco (LAFEPE), Recife, Brazil and Laboratorio ELEA, Ciudad Autónoma de Buenos Aires, Argentina), which have trypanocidal activity against all parasitic forms. Because of the side effects, which can interrupt the therapeutic protocol,^10^ and limited cure efficacy (acute phase, 50–80%; chronic phase, 8–20%), they are considered far from ideal.^11–16^ In addition to the CD phase, other factors influencing the cure efficacy of both drugs include the treatment period, dose, age and immune system of patient, and geographical patient origin.^11^ Moreover, the existence of *T. cruzi* strains naturally resistant to both drugs may partly explain the low cure rates detected in treated chagasic patients.^17^

According to the World Health Organization (WHO), the ideal drug for CD treatment should have the following characteristics: parasitological cure during the acute and chronic phases, efficacy in one dose or a few doses, low cost, absence of side effects or teratogenic effects, and no induction of resistance. However, no drug meets all these requirements then new, more effective, and better-tolerated compounds are urgently needed.

In the present review, we analyze the current data regarding the development of new drugs for CD and discuss current treatments, clinical trials, and the testing of new compounds. In addition, we review the pharmacokinetics and biodistribution of BZ.

## METHODS

We searched the Medline database for articles published in English from 1952 to 2017 using the terms “Chagas disease”, “benznidazole”, and “nifurtimox”. Ongoing and completed clinical trials were queried at ClinicalTrials.gov. Google was used for additional queries of specific references freely available on the internet.

### Current drugs used for the treatment of Chagas disease.

#### Nifurtimox.

NFX was the first drug used for CD treatment. Packchanian^18^ was the first to experimentally demonstrate that nitrofurans were promising for CD treatment. Later, Brener^19^ used nitrofurazone to cure chronically infected mice. Although important results were reported regarding treatment with nitrofurazone,^20–23^ the unfavorable side effects and toxicity ceased its use. Clinical trials with NFX started in 1965 in South America, and the results differed based on the disease phase, treatment duration, patient age, and geographical area, with the best results obtained during the acute phase in children and patients with a recent infection (8–10 mg/kg/day for 60–90 days)^10,11^; negative xenodiagnosis was achieved in 88–100% of acute phase patients who completed the treatment schedule. The treatment effectiveness in adult patients with chronic disease was low, with a cure rate of 7–8% in the chronic indeterminate phase; however, in children < 14-years old in the chronic asymptomatic phase, the cure rate was significantly higher, reaching up to 85.7%.^11,24^ Table 1 describes the studies in which NFX was used to treat CD.

**Table 1:** Summary of the studies in which nifurtimox was used to treat Chagas disease

Reference no.	Year	Country	No. of patients	Age (years)*	Treatment protocol	Follow-up*	Results at the end of the study
25	1977	Argentina	42	Not shown	8–10 mg/kg 60–120 days	≥ 12 months	27/29 negative XD
25	1977	Chile	15	Not shown	8–10 mg/kg 60–120 days	≥ 12 months	12/14 negative XD
25	1977	Brazil	52	Not shown	8–10 mg/kg 60–120 days	≥ 12 months	35/44 negative XD
26	1990	Brazil	50	Not shown	10–15 mg/kg 60–120 days	2 years	50% negative XD, 6% negative serology
27	1990	Argentina	39	< 17	8–10 mg/kg 60 days	139 months	11–14% negative serology, 15% negative XD
28	1997	Brazil	27	Not shown	5 mg/kg 30 days	1 year	100% positive serology, 8/83 positive XD
29	1998	Chile	28	< 10	7 mg/kg 60 days	6 months	100% negative XD, 35.8% negative PCR
30	2000	Argentina	32	13–52	5–8 mg/kg 60 days	14 years (8–23)	100% positive serology, 100% negative XD
31	2000	Brazil	28	Adults	10 mg/kg 60 days	10 years	100% positive serology, 100% positive PCR
32	2001	Chile	66	Children	Not shown	3 years	34/36 positive serology, 100% negative XD and PCR
33	2002	Brazil	10	38 (25–48)	8–9 mg/kg 60 days	303 months	100% positive serology, 9/10 positive XD
34	2003	Chile	99	Children	10 mg/kg 30 days	3 years	100% negative XD, 100% negative PCR
24	2004	Argentina	7	< 14	12–15 mg/kg 45–60 days	21 years (median)	6/7 negative serology
35	2013	Chile	21	38 (23–50)	6 mg/kg 60 days	13 months	Four patients positive PCR†
36	2013	Switzerland	37	44 (22–59)	10 mg/kg 30–60 days	4 years	100% positive serology, one patient positive PCR

PCR = polymerase chain reaction; XD = xenodiagnosis.

* The data are expressed as mean except where noted otherwise. In some instances, the range is shown in parentheses.

† PCR was performed in both the patient’s blood and fecal samples of *Triatoma infestans* nymphs.

The most frequent side effects are anorexia, weight loss, paresthesia, drowsiness or psychic excitability, and gastrointestinal symptoms such as nausea, vomiting, and occasional intestinal cramps. Treatment with NFX, even at low doses, has more intense side effects than BZ; a high number of treatment attempts were interrupted because of severe digestive intolerance.^28^ Incomplete treatment was recently shown to lead to NFX resistance.^37^

#### Benznidazole.

Near the end of the 1970s, Grunberg et al.^38^ showed for the first time that BZ was active against *T. cruzi*. BZ was shown to have similar efficacy as nitrofurazone in both the acute and chronic phases but with less toxic effects.^39^ Thereafter, several experimental and clinical CD treatments using BZ were published.^40–43^ Numerous clinical studies showed that BZ had significant activity during the acute phase (all parasitological and conventional serological tests had up to 80% negative results).^14,44^ Although several reports have demonstrated its effectiveness, the major limitation of BZ is the low cure rate during the chronic phase. In 2002, Cançado^15^ observed cure in 76% of patients with acute phase CD (13–21-year follow-up) and only 8% of patients with chronic phase CD (6–18-year follow-up), supporting previous studies demonstrating the lack of effect during the chronic phase. Table 2 describes the studies in which BZ was used to treat CD.

**Table 2:** Summary of the studies in which benznidazole was used to treat Chagas disease

Reference no.	Year	Country	No. of Patients	Age*	Treatment protocol	Follow-up*	Results at the end of the study
45	1994	Argentina	131	9–66	5 mg/kg 30 days	5–13 years	21/110 negative serology, 18/18 negative XD
46	1996	Brazil	64	7–12	7.5 mg/kg 60 days	36 months	37/64 negative serology
28	1997	Brazil	50	Not shown	5 mg/kg 30 days	12 months	24/26 negative XD
47	1998	Argentina	106	6–12	5 mg/kg 60 days	48 months	27/44 negative serology, 40/42 negative XD
30	2000	Argentina	36	13–52	5 mg/kg 30 days	14 (8–23) years	100% positive serology, 100% negative XD
31	2000	Brazil	17	Adults	10 mg/kg 60 days	10 years	100% positive serology, 100% positive PCR
48	2000	Argentina	130	33 (10–79)	4–8 mg/kg 45–60 days	80 months	3/130 negative serology, 3/46 negative PCR
15	2002	Brazil	113	9–69	5–10 mg/kg 40–60 days	6–18	9/113 negative serology
24	2004	Argentina	64	< 14	5 mg/kg 30 days	13 years (median)	23/37 negative serology
49	2006	Argentina	283	39 (30–50)	5 mg/kg 30 days	9.8 years	32/218 negative serology
50	2006	Brazil	27	49 (23–88)	5 mg/kg 60 days	24 months	24/27 negative blood culture
51	2007	Argentina	27	17–46	5 mg/kg 45–60 days	20.6 years	9/27 negative serology, 100% negative XD
52	2009	Honduras	232	< 12	5–7.5 mg/kg 60 days	36 months	215/232 negative serology
52	2009	Guatemala	124	< 15	5–7.5 mg/kg 60 days	18 months	18/31 negative serology
52	2009	Bolivia (Entre Ríos)	1,409	< 15	5–7.5 mg/kg 60 days	60 months	42/1007 negative serology
52	2009	Bolivia (Sucre)	1,040	< 18	5–7.5 mg/kg 60 days	9–27 months	0 negative serology
53	2014	Spain	26	40	300 mg/day 60 days	10 months	100% positive serology, 16/17 sustained negative PCR
54	2015	Colombia, El Salvador, Brazil, Argentina	1,431	55 (44–61)	300 mg/day 40–80 days	7 years	59.5% PCR+ after treatment

PCR = polymerase chain reaction; XD = xenodiagnosis.

* The data are expressed as mean values except where noted otherwise. In some instances, the range is shown in parentheses.

Drug efficacy is dependent on the susceptibility of different *T*. *cruzi* strains to the compound.^41,55,56^ The existence of strains that are naturally resistant to BZ and NFX has been previously described and poses a major challenge to the development of new anti-*T. cruzi* drugs.^56^ Geographical differences of *T. cruzi* strain might affect cure efficacy because of parasite genetic variability. More than 80% of CD patients in the acute or chronic phase from Chile, Argentina, and Southern Brazil (state of Rio Grande do Sul) treated with NFX showed a high percentage of cure based on xenodiagnosis and serology.^28^ By contrast, only 40% of cure was detected in the treated patients with CD from Brazilian states of São Paulo, Minas Gerais, Bahia, and Goiás.

Patient age is also an important factor for BZ efficacy. In children with CD aged 6–12 years, treated with BZ for 60 days had a cure efficacy of approximately 56%^46^ and 62%,^47^ similar to children aged 6–12 years with an indeterminate CD phase in Argentina,^47^ in which negative seroconversion was observed in 62% of the treated group.

According to current recommendations for CD treatment from the I Latin American Guidelines for the Diagnosis and Treatment of Chagas Heart Disease,^57^ BZ chemotherapy is indicated for children, acute cases (congenital transmission included), laboratory accidents, and reactivation (pharmacologically immunosuppressed and human immunodeficiency virus (HIV)-infected patients). In adult patients with an indeterminate phase or established chronic chagasic cardiomyopathy, indications for parasite treatment remain controversial.^57^ However, in the largest recent study^49^ to show that BZ treatment slows the development and progression of cardiomyopathy in adults with chronic infection,^49,51^ 566 adults with chronic infection but without advanced heart disease were chosen to receive BZ or no treatment. Significantly fewer treated patients showed disease progression or electrocardiographic (ECG) abnormalities despite seroconversion in only 15% of these patients (median follow-up, 9.8 years).^49^

A recent retrospective study has shown that treatment with BZ prevents the occurrence of ECG alterations and decreases serological immunofluorescence titers in patients with chronic CD.^58^

The most frequent adverse effects observed with BZ are skin manifestations, paresthesia, peripheral neuropathy, anorexia, and weight loss; decreased bone marrow, thrombocytopenic purpura, and agranulocytosis are the most severe manifestations.^10,59^ Side effects have led to treatment interruption in approximately 12–13% of patients.^18,30,49^ However, other studies have found higher levels of treatment interruption, in 25%^60^ and 41.5%^61^ of patients. These differences might be attributable to the treatment duration (30 days in the former and 60 days in the latter studies).

#### Mechanisms of action of nifurtimox and benznidazole.

**Figure 1.**
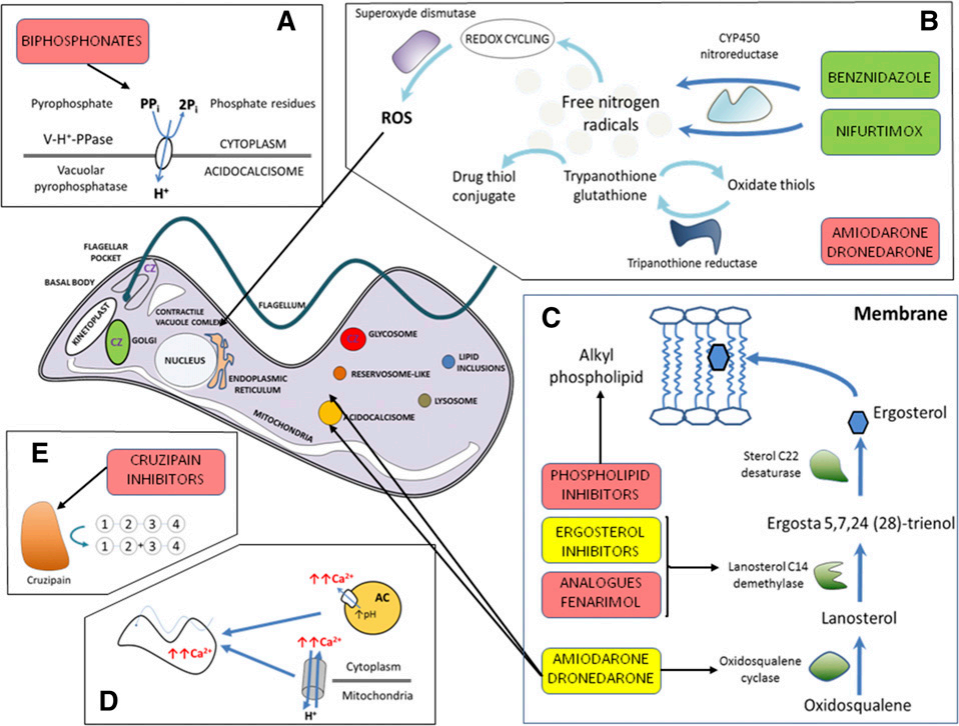
Schematic representation of the mode of action of the major drugs with trypanocidal activity. The green boxes represent drugs currently used for treatment, the yellow boxes represent drugs in clinical trials, and the red boxes represent experimental drugs. (**A**) Bisphosphonates inhibit farnesyl pyrophosphate synthase, which reduces the levels of sterols and other essential poly-isoprenoids compounds, affecting cell viability. (**B**) Nifurtimox (NFX) and benznidazole (BZ) are reduced by the parasite nitroreductase, resulting in the production of reactive oxygen species (ROS), which directly damage the cells of the parasite. Trypanothione reductase helps relieve the oxidative stress, and inhibitors of this enzyme, such as thioridazine and sulfoximine buthionine (SB), increase the amount of ROS in the intracellular space. (**C**) The ergosterol biosynthetic pathway is essential for parasite survival. Blocking this pathway leads to loss of cell viability via depletion of essential sterols and accumulation of toxic intermediates. Ergosterol inhibitors and fenarimol analogues target lanosterol C14 demethylase, and amiodarone and dronedarone partially inhibit oxidosqualene cyclase. Phospholipid inhibitors block sterol synthesis, inhibit de novo phospholipid synthesis via Greenberg’s pathway, and inhibit signal transduction enzymes such as phosphatidylinositol phospholipase C. (**D**) Amiodarone and dronedarone release Ca^2+^ from mitochondria and acidocalcisomes (ACs), which increases Ca^2+^ levels in the cytoplasmic space and compromises cell survival. (**E**) Cruzipain (CZ) is typically located in the Golgi apparatus, flagellar pocket, and glycosomes and is an essential cysteine protease involved in parasite differentiation, cell invasion, multiplication, and immune evasion. Inhibitors of cruzipain alter the Golgi apparatus, owing to the accumulation of unprocessed cruzipain precursors. This figure appears in color at www.ajtmh.org.

Figure 1 shows the mechanism of action of NFX, BZ, and other trypanocidal drugs.

The mechanisms of action of BZ and NFX are not entirely clear. BZ reportedly acts via reductive stress involving covalent modification of macromolecules such as DNA, proteins, and lipids.^62^ In addition, BZ and its metabolites can affect the trypanothione metabolism of *T. cruzi*.^63^ BZ also improves phagocytosis,^64^ increases trypanosomal death through IFN-γ induction,^64^ and inhibits *T. cruzi* NADH-fumarate reductase.^65^

The reduction of NFX to a nitro anion radical followed by the autoxidation of this radical produces highly toxic oxygen metabolites.^62^ Its deficient metabolic detoxification mechanisms for oxygen render *T. cruzi* highly susceptible to partial reduction products of oxygen, particularly hydrogen peroxide; therefore, it is more sensitive to oxidation than the vertebrate cells.^62,66^

Previous studies suggest that the nitroheterocyclic compounds BZ and NFX are prodrugs and require activation by nitroreductases for cytotoxic activity.^67^ Interestingly, the deletion of copies of genes encoding two different nitroreductases, namely, old yellow enzyme (TcOYE, also named prostaglandin synthase)^68^ and trypanosomal type I nitroreductase (NTR-1),^67^ has been associated with the resistance of *T. cruzi* to NFX and BZ in vitro. A functional analysis associated reduced NTR-1 levels in *T. cruzi* and *T. brucei* with resistance to nitroheterocyclic compounds, whereas overexpression of this enzyme resulted in hypersensitivity.^67^ NTR-1 is absent from mammals, is selective, and catalyzes the two-electron reduction of nitroheterocyclic compounds within the parasite, producing toxic metabolites.^69^ Interestingly, a recent study using a metabolomic analysis showed that the covalent binding of BZ with thiols as well as protein thiols is the major mechanism of BZ toxicity against *T. cruzi* metabolites.^70^ Although the mechanism of drug resistance in this parasite remains poorly understood, differences in susceptibility to BZ and NFX between *T. cruzi* strains^17,56,71^ and/or the genetic diversity of the host^56^ might explain, in part, the variations in the efficacies of these antiparasitic drugs.

In addition to its effects on *T. cruzi*, nitroreductive bioactivation is also responsible for mammalian BZ toxicity because of the interaction between its reactive metabolites and DNA, proteins, lipids, and other relevant cellular components.

### Clinical trials and other studies for Chagas disease treatment.

Since the introduction of BZ and NFX, only allopurinol and the azoles itraconazole, fluconazole, ketoconazole, posaconazole (POSA), and ravuconazole (RAVU) have been studied in clinical trials, observational studies, or clinical cases.^53,72–76^ When designing novel drugs, specific targets of *T. cruzi* should be identified using cellular and molecular approaches to achieve both high efficacy and low toxicity.^77,78^ Currently, the main experimental/preclinical approaches for anti-*T. cruzi* drugs are based on the inhibitors of ergosterol, trypanothione metabolism, cysteine protease, pyrophosphate metabolism, protein and purine synthesis, lysophospholipid analogues (LPAs), and natural drugs.^79^ Unfortunately, only a few clinical trials for CD treatment are ongoing or were performed recently (Table 3).

**Table 3:** Current status of the drugs used to treat Chagas disease

Drug	Drug development	In vitro assay	In vivo Assay	Phase I studies	Phase II studies	Phase III studies	Phase IV/approved
BZ	✓	✓	✓	✓	✓	✓	✓
NFX	✓	✓	✓	✓	✓	✓	✓
POSA	✓	✓	✓	✓	✓	–	–
RAVU	✓	✓	✓	✓	✓	–	–
ITRA	✓	✓	✓	✓	✓	–	–
KETO	✓	✓	✓	✓	X	–	–
VORI	✓	✓	✓	✓	–	–	–
ALBA	✓	✓	✓	✓	–	–	–
DO8701	✓	✓	✓	–	–	–	–
TAK-187	✓	✓	✓	–	–	–	–
K-777	✓	✓	✓	X	–	–	–
FENARI	✓	✓	✓	Planned	–	–	–
FEXINI	✓	✓	✓	✓	✓	–	–
MILTEFO	✓	✓	✓	✓	–	–	–
EDELFO	✓	✓	–	–	–	–	–
ILMOFO	✓	✓	–	–	–	–	–
NANO BZ	✓	✓	✓	–	–	–	–
SELENIUM	✓	✓	✓	✓	✓	In progress	–
ALOPU	✓	✓	✓	✓	X	–	–
AMIO	✓	✓	✓	✓	In progress	–	–
SCYX-7158	✓	✓	✓	In progress	–	–	–

ALBA = albaconazole; ALOPU = allopurinol; AMIO = amiodarone; BZ = benznidazole; EDELFO = edelfosine; FENARI = fenarimol; FEXINI = fexinidazole; ILMOFO = ilmofosine; ITRA = Itraconazole; KETO = ketoconazole; MILTEFO = miltefosine; NANO BZ = benznidazole nanoformulated; NFX = nifurtimox; POSA = posaconazole; RAVU = ravuconazole; SCYX-7158 = oxaborole; VORI = voriconazole; X = interrupted.

#### Ergosterol biosynthesis inhibitors.

##### Posaconazole.

POSA (SCH 56592; Schering-Plough Research Institute) is a potent and selective inhibitor of fungal and protozoan CYP51, a cytochrome P-450 family member. It is commercially available for the prophylaxis of invasive fungal infections and treatment of azole-resistant candidiasis.^80^ POSA also has potent trypanocidal activity in vitro^81^ and in vivo^82,83^ against *T. cruzi* strains naturally resistant to nitrofurans, nitroimidazoles, and conventional antifungal azoles.^83^ More importantly, POSA was more active than the reference drug, BZ, against drug-resistant *T. cruzi* strains in murine models of acute and chronic CD.^83^ However, recent studies have demonstrated an advantage of BZ over POSA. In an in vitro study comparing the activity of nitroheterocyclics with the activity of POSA and RAVU against intracellular *T. cruzi* amastigotes representing all current discrete typing units (DTUs), the nitroheterocyclics showed broad, but less potent, efficacy against all *T. cruzi* DTUs tested, whereas POSA and RAVU showed variable activity and were unable to eradicate intracellular infection even after 7 days of continuous compound exposure.^84^ In an in vivo study, POSA failed as single treatment and in combination with BZ to clear an infection with a BZ-resistant strain and was less effective in curing infections with BZ susceptible strains.^85^

The success demonstrated with POSA in a patient with chronic CD and systemic lupus erythematosus^86^ encouraged the initiation of two phase II clinical trials in CD patients (ClinicalTrials.gov Identifiers: NCT01377480 and NCT01162967). One of these clinical trials (CHAGASAZOL) concluded in August 2012 and was an independent study financed by the Spanish Ministry of Health and performed by Vall d’Hebron University Hospital and the International Health Program of the Catalan Health Institute (PROSICS).^53^ This multicenter, randomized, open-label clinical trial compared BZ (5 mg/kg/day for 60 days) and two schedules of POSA (100 mg/12 hours and 400 mg/12 hours for 60 days) in 78 chronic CD patients. During the follow-up, a greater proportion of patients had treatment failure with POSA than BZ, as measured by positivity with real-time PCR of *T. cruzi* in peripheral blood. The other study, STOP CHAGAS (ClinicalTrials.gov Identifier: NCT01377480), was completed in 2015. The successful response, which was defined as a negative qualitative PCR value at the day 180 follow-up were the following: POSA (13.3%), Placebo (10%), POSA + BZ (80%) and BZ + Placebo (86.7%); *P* = 0.69 for POSA versus Placebo; *P* < 0.0001 for POSA versus POSA + BZ. These data reinforce the idea that BZ monotherapy is superior to POSA either as monotherapy or as combination therapy.^87^

In addition, POSA is an extremely expensive drug, and its cost can hinder its use in developing countries.^88^

##### Ravuconazole.

It is a triazole derivative with potent and broad-spectrum antifungal activity. In murine models of acute CD, RAVU had high parasitological cure activity against nitrofuran/nitroimidazole-susceptible (CL strain) and partially drug-resistant (Y strain) *T. cruzi* strains, but no curative activity in mice infected with the fully drug-resistant Colombiana strain in a model of chronic CD.^89^ In a canine model of acute CD, RAVU had potent suppressive, but not curative, activity.^90^ The short terminal half-life of RAVU in mice (4 hours) and dogs (8.8 hours) may explain these results. The longer half-life in humans (4–8 days) encouraged its use for chemotherapy in human CD. One advantage is the need for less frequent use of RAVU than BZ and NFX.

The major advantages of RAVU include its simpler chemical structure and low price compared with POSA.^91^ In 2009, the Drugs for Neglected Diseases initiative (DNDi) collaborated with Eisai Co. Ltd., a Japanese pharmaceutical company that discovered E1224, to develop a new chemical entity for CD. E1224 is a prodrug that converts to RAVU, leading to improved drug absorption and bioavailability.^76^ A phase II randomized, multicenter, placebo-controlled study evaluated the safety and efficacy of three oral E1224 dosing regimens (high dose for 4 or 8 weeks; low dose for 8 weeks) and BZ (5 mg/kg/day) in 231 adult patients with chronic indeterminate CD who were recruited from research centers in Tarija and Cochabamba, Bolivia (ClinicalTrials.gov Identifier: NCT01489228). E1224 showed good safety and was effective in clearing the *T. cruzi*, but 1 year after treatment, only 8–31% of patients treated with E1224 maintained parasite clearance compared with 81% of BZ-treated patients, demonstrating that E1224 has low parasite eradication rates.^76^

##### Itraconazole.

Itraconazole, a synthetic imidazole derivative, has shown good efficacy against *T. cruzi* both in vitro and in vivo.^92^ In a blinded study of itraconazole use (6 mg/kg/day for 120 days) in 46 patients with chronic CD from an endemic area of Chile, monitoring for ECG abnormalities and xenodiagnosis or real-time xenodiagnosis quantitative polymerase chain reaction for *T. cruzi* infection was conducted before treatment and annually for 20 years.^93^ The control group consisted of 67 patients with chronic indeterminate CD who were followed-up for 4 years, and for ethical reasons, this group was treated after the experimental period. After the 20 years, only 10.86% of the patients had developed ECG abnormalities, and 32.6% had negative xenodiagnosis test results, indicating that itraconazole prevents ECG abnormalities. The major limitation of this study is that xenodiagnosis and PCR are not reliable indicators of cure because they show low sensitivity for *T. cruzi* detection in chronic CD patients.^14^

#### Amiodarone/Dronedarone.

Amiodarone is a class III antiarrhythmic agent frequently used for the treatment of symptomatic patients with the clinical cardiac form of CD. It has direct activity against *T. cruzi*, both in vitro and in vivo, and potent synergistic activity with POSA.^94^ In addition to disrupting Ca^2+^ homeostasis in *T. cruzi* by inducing Ca^2+^ release from intracellular stores, specifically the single giant mitochondrion, amiodarone also blocks ergosterol biosynthesis.^94^ Treatment with amiodarone was associated with clinical improvement in at least one clinical case of human CD.^95^ An observational study showed that cardioverter-defibrillator implantation plus amiodarone reduced the risk of all-cause mortality and sudden death compared with amiodarone alone in chagasic patients with heart disease and life-threatening ventricular arrhythmias.^96^ Currently, one clinical trial with amiodarone is ongoing (ClinicalTrials.gov Identifier: NCT01722942), in which the efficacy of an implantable cardioverter defibrillator is compared with that of amiodarone in the primary prevention of all-cause mortality in high-risk patients with chagasic cardiomyopathy and nonsustained ventricular tachycardia; the estimated study completion date is 2019.

The main advantage of amiodarone is its dual role; it is a commonly used antiarrhythmic drug as well as a potent and selective anti-*T. cruzi* agent.^97^ More recently, potent anti-*T. cruzi* activity was demonstrated in vitro with dronedarone, an amiodarone derivative designed to eliminate the thyroid toxicity frequently observed with amiodarone treatment; importantly, the 50% inhibitory concentrations against parasites were lower than those previously reported for amiodarone. These results suggest a possible future repurposing of dronedarone for CD treatment.^98^

#### Benznidazole-new clinical trials.

##### The BENEFIT.

The most important study in recent years was the BENEFIT project (Benznidazole Evaluation for Interrupting Trypanosomiasis; ClinicalTrials.gov Identifier: NCT00123916), which was a multicenter, double-blind, placebo-controlled trial of trypanocidal treatment of BZ for patients with chronic Chagas heart disease in 54 study centers in Argentina, Bolivia, Brazil, Colombia, and El Salvador. The objective was to evaluate the effect on the clinical progression of Chagas’ cardiomiopathy (mortality and other major cardiovascular clinical outcomes in patients with chronic Chagas heart disease). Moreover, it was intended to investigate whether etiologic treatment significantly could reduce parasite burden, as assessed by PCR-based techniques, and to determine the safety and tolerability profiles of the trypanocidal drug in this chagasic population.^99,100^ BZ was administered as a fixed daily dose of 300 mg for 40–80 days; the time period was adjusted according to body weight, with a total minimum dose of 12 g (corresponding to 40 kg) and total maximum dose of 24 g (corresponding to 80 kg). BZ was able to reduce significantly the detection of parasites in the circulation, but they failed to demonstrate a reduction in the progression of cardiomyopathy in the overall chagasic patients.^54^ However, a recent retrospective study has shown that the treatment with BZ prevents the occurrence of ECG alterations and decreases serological immunofluorescence titers in patients with chronic CD.^58^ The different follow-up period (5.4 years and two decades for BENEFIT and for the retrospective study, respectively) and the clinical manifestation (patients with established heart disease in BENEFIT and patients with a previous normal ECG in the retrospective study) may explain this discrepant outcome between the BENEFIT assay and the latter as well as other studies such as the ones performed by Viotti and coworkers.^17^

##### The TRAENA.

The TRAENA (treatment in adult patients; TRAtamiento EN pacientes Adultos [in Spanish]) study is a clinical, randomized, double-blind, phase III study conducted at Instituto Nacional de Parasitología “Dr. Mario Fatala Chaben” and aimed at determining if BZ is capable of changing the natural evolution of chronic CD in adult patients (ClinicalTrials.gov Identifier: NCT02386358). Treated patients will be followed-up for 7–11 years after treatment. Because the study currently remains blind, the present serum and parasitology data refer to the total patient population, irrespective of assignment (BZ or placebo).^101^

##### Pediatric formulation.

Until recently, the only formulation available for children (Laboratorio ELEA) was not ideal because it provided 50 mg BZ per tablet. Therefore, a phase IV study sponsored by DNDi in collaboration with Pernambuco State Pharmaceutical Laboratory (LAFEPE) in Brazil was launched to respond to the need for an age-adapted and easy-to-use pediatric formulation^102^ (ClinicalTrials.gov Identifier: NCT01549236) for children weighing < 20 kg (12.5 mg BZ per tablet). All children showed complete parasitic clearance after treatment and 12 months later. Importantly, the children had lower blood levels of parasites than previously documented in adults. The pediatric formulation was registered in Brazil in 2011 and was included on the WHO’s Essential Medicines List for children in 2013.

After the formulation was available, a second objective was describing the pharmacokinetic parameters of BZ in children with acute or early chronic indeterminate CD. Another clinical trial (ClinicalTrials.gov Identifier: NCT00699387) with pediatric CD patients demonstrated lower BZ concentrations in children < 7-years old compared with older children and adults, indicating more rapid BZ elimination in the former. Nevertheless, the efficacy of cure was equal or better in patients < 7-years old, and the children had few adverse reactions to the drug.^103^

##### New benznidazole regimens/combos.

A project led by DNDi has as objective the development of a new BZ and RAVU (E1224) combination treatment regimen for chronic CD.^104^ A Phase I drug-drug interaction study assessed the safety and pharmacokinetics interaction of E1224 and BZ administered first separately and then in combination in healthy human volunteers, and no major clinically relevant safety or tolerability issues were identified. So, a proof-of-concept (PoC) evaluation of new treatment regimens of BZ in monotherapy or in combination with E1224 (now denominated fosravuconazole) will be assessed versus placebo, for the treatment of adult patients with chronic CD and recruitment started by the end of 2016.^104^ The objective is to determine if the safety and tolerability issues of BZ can be managed by reduced doses and treatment duration.

### Nifurtimox-new clinical trials.

A phase I study (ClinicalTrials.gov Identifier: NCT01927224) sponsored by Bayer, with chronic CD patients aged 18–45 years, was launched in 2013 to evaluate the bioequivalence, safety, and tolerability of a novel 30 mg NFX tablet compared with the marketed 120-mg tablet when administered after a high-fat/high-calorie test meal. This study aimed to develop an age-appropriate pediatric oral dosage for CD treatment. This clinical trial, completed in 2014, showed that the new 30-mg NFX oral tablet formulation was bioequivalent to the marketed 120 mg NFX. In the absence of differences in clinically relevant safety findings, the pharmacokinetics data show that the 30-mg tablet is a viable formulation for administration of NFX in children. Thereafter, a second clinical trial, phase I, required as part of the clinical development of an age appropriate pediatric oral dosage form, was launched to evaluate the effect of food on the absorption of the drug as well as the safety and tolerability of the novel 30 mg in adults suffering from chronic CD when administered after a high-fat/high-calorie test meal compared with a fasting state (ClinicalTrials.gov Identifier: NCT02606864).

The CHICAMOCHA 3-Equivalence of Usual Interventions for Trypanosomiasis is a randomized, blind, parallel-group phase II/III trial that will investigate if NFX is an effective trypanocidal agent (by comparison with placebo) and equivalent to BZ in terms of both parasite-related and safety outcomes in patients with CD from Colombia and Argentina without clinical signs of dilated cardiomyopathy (ClinicalTrials.gov Identifier: NCT02369978). The estimated study completion date is 2017.

#### Selenium.

Selenium supplementation decreases heart damage during acute^105^ and chronic *T. cruzi* infection^106^ in murine experimental models, by protecting the heart from inflammatory damage without protection from infection.^105^ Moreover, a positive correlation between cardiac insufficiency and low selenium levels in patients with advanced chronic chagasic cardiomyopathy has been observed.^107^

Based on these studies, the Oswaldo Cruz Foundation and Conselho Nacional de Desenvolvimento Científico e Tecnológico launched a phase III study, the Selenium Treatment and Chagasic Cardiopathy study (ClinicalTrials.gov Identifier: NCT00875173), with an estimated study completion in 2020. By studying the rate of progression and comparing ventricular ejection fractions, this clinical trial is investigating whether oral selenium treatment can reduce the progression of heart dysfunction in chagasic patients. The primary and secondary endpoints are a 50% reduction in the progression rate of heart dysfunction and partial or total reversion of electrocardiography alterations, respectively.

#### New nitroimidazoles.

Fexinidazole is a 5-nitroimidazole with potent trypanocidal activity that has been rediscovered through extensive compound mining by DNDi. It can induce high levels of parasitological cure in mice infected with BZ-susceptible, partially resistant and resistant *T. cruzi* strains in acute and chronic experimental CD, which is an important improvement compared with the current standard treatment with BZ.^108^ Importantly, a recent study demonstrated that sulfoxide and sulfone fexinidazole metabolites were more effective than either fexinidazole itself or BZ in treating mice acutely infected with the partially resistant *T. cruzi* Y strain.^109^ Fexinidazole is already in phase II and phase III clinical development for human African trypanosomiasis (ClinicalTrials.gov Identifier: NCT01685827). Recently, a phase II PoC study sponsored by DNDi was launched to evaluate if the treatment with fexinidazole will lead to a better sustained clearance of the parasites at 12 months of follow-up in patients with chronic indeterminate CD (ClinicalTrials.gov Identifier: NCT02498782). High efficacy rates of fexinidazole encouraged the design of a new PoC study that will be started in 2017.^110^

### Experimental new drugs.

Drug development for tropical diseases has largely relied on three strategies: label extension, piggyback discovery, and de novo drug discovery.^111^ Label extension consists of extending the indications of existing treatments for other human and animal illnesses to tropical diseases. Piggyback discovery is used when a molecular target present in parasites is being pursued for other (commercial) indications to facilitate the identification of chemical starting points. De novo drug discovery relies on the identification of new chemical entities as novel antiparasitic drugs and is more long-term and expensive than the other approaches.^111^

Advances in knowledge of the metabolic pathways of *T. cruzi*, including the differences in the metabolism of parasite and mammalian cells,^11,112–114^ have allowed the identification of rational targets for the development of safe and more effective drugs for CD treatment. Murine models have been used to evaluate the therapeutic efficacy of different agents during infection with *T. cruzi*. Currently, protein targets are being selected for the development of new anti-CD drugs because of their direct involvement in the survival and replication of the parasite, as well as in disease progression. Some compounds have trypanocidal activity in vitro and in vivo,^115^ further reducing inflammation and subsequent tissue damage.^116,117^

#### Inhibitors of trypanothione metabolism.

Several studies have identified the enzymes involved in trypanothione metabolism as potential chemotherapeutic targets.^118,119^ Trypanothione reductase (TR) plays an essential role in the life of *T. cruzi* because it maintains the intracellular reducing environment.^119^ This biochemical pathway is unique to kinetoplastid protozoa. The structural differences between TR and its human counterpart glutathione reductase also make TR a promising target; whereas glutathione reductase has a narrow, positively charged active site to accommodate the glycine carboxylates of its substrate glutathione, TR has a wider, noncharged, and more hydrophobic active site.^120^ Inhibitors of the TR pathway have recently received the attention of many research groups, and a variety of compounds have been identified as TR inhibitors.^121–124^ Thioridazine, an in vitro TR inhibitor,^121^ increased survival and reduced parasitemia and cardiac injury in murine models of acute infection,^125,126^ although parasitological cure was not observed. Inhibitors of trypanothione metabolism, such as sulfoximine buthionine, are potential candidates, either alone or combined with drugs that produce free radicals such as NFX and BZ.^114^ Although promising, more than 20 years of research have failed to identify any feasible clinical candidates from these classes of compounds.

#### Inhibitors of cysteine proteases.

Cruzain, also known as cruzipain, is the major cysteine protease (gp57/51) of *T. cruzi* and is expressed in all developmental forms of different *T. cruzi* isolates.^127,128^ Protease inhibitors blocked amastigote and epimastigote proliferation and metacyclogenesis in vitro, significantly reduced parasitemia, and increased animal survival in a murine model in both CD phases.^129^ In the CD acute phase, treatment of mice with irreversible cruzain inhibitors reduced the number of cardiac lesions and intracellular amastigotes and the levels of inflammatory infiltrates. Although it is a good target, its short half-life needs large doses and continuous administration to achieve its effect.

K-777, which was originally characterized by the Sandler Center for Research in Tropical Parasitic Disease at the University of California, San Francisco, was the most promising cysteine protease inhibitor. It is a vinyl sulfone that effectively blocks cruzain activity and has rescued mice from the acute phase of a lethal experimental *T*. *cruzi* infection and cleared parasitemia in chronically infected mice without toxicity to the mammalian host.^128^ It promotes the accumulation of unprocessed cruzain precursor molecules in the Golgi cisterns, leading to parasite death.^129^ In an acute model of infection in dogs, K-777 did not promote parasitological cure but significantly reduced parasite-induced heart damage.^117^ Preclinical safety and toxicology studies were performed to complete the investigational new drug package for clinical evaluation of K-777 for CD treatment. Unfortunately, the development of this compound was interrupted because of tolerability findings at low dose in primates and dogs.^130^

#### Lysophospholipid analogues (LPAs).

LPAs were designed approximately four decades ago as both potential immunomodulators and antimetabolites of phospholipid metabolism.^131^ The alkyl-lysophospholipids are synthetic derivatives of LPAs that comprise a new class of compounds that are promising for chemotherapy of diseases caused by kinetoplastids and have in vitro and in vivo effects against *T. cruzi* strains that are susceptible (Tulahuen strain), partially resistant (Y strain), and naturally resistant to nitrofurans/nitroimidazoles (Colombiana strain).^132–134^ Several studies have described their mechanism of action against *T. cruzi*.^135,136^ In murine models, the alkyl-lysophospholipids ilmofosine, miltefosine (hexadecylphosphocholine), and edelfosine had suppressive activity but did not cure the infection with *T. cruzi* Y strain, and ilmofosine and miltefosine had suppressive activity but did not cure the infection with *T. cruzi* Tulahuen strain.^132^ In another murine study, miltefosine promoted survival and reduced the parasitemia of Y strain-infected mice to levels as effectively as BZ. Four months after treatment, no parasites were detected in the blood or spleen tissue sections maintained in culture; however, more sensible methods to assess a cure were not used.^133^ The development of LPAs as anti-cancer agents enables knowledge of their pharmacology, toxicology, and tolerance in humans and reduces the drug development cost for tropical diseases.^132^

#### Ergosterol biosynthesis inhibitors.

##### TAK-187.

TAK-187 is a triazole with potent activity against *T. cruzi* in vitro and in vivo. In a murine model of acute CD using *T. cruzi* strains with different susceptibilities to the currently available drugs, TAK-187 treatment resulted in complete protection against death and high levels (60–100%) of parasitological cure against all strains. In chronic disease models, TAK-187 resulted in 80–100% survival, with parasitological cure in 80–100% of survivors.^137^ Importantly, no toxic side effects were observed in any of the experimental protocols. Another study has demonstrated that TAK-187 is more effective than BZ in preventing cardiac damage in experimental CD.^138^

##### D0870.

The bis-triazole derivative D0870 has in vivo activity against a variety of *T. cruzi* strains, including nitroimidazole/nitrofuran-resistant strains, in both acute and chronic disease murine models.^139^ D0870 treatment cured 30–45% of chronic infections with various strains in animals, including the Colombiana strain, whereas no cure was obtained with BZ. Importantly, the trypanocidal activity of D0870 was largely retained even in immunosuppressed hosts. Unfortunately, AstraZeneca, a proprietary biopharmaceutical company, interrupted the development of this compound because D0870 promoted QT prolongation at modest serum concentrations and led to adverse cardiac events in a HIV-positive patient receiving the drug for fluconazole-resistant oropharyngeal and esophageal candidiasis.^140^

##### Albaconazole.

Albaconazole (UR-9825; Uriach y Cia) is an experimental triazole derivative with potent and broad-spectrum antifungal activity. Its in vitro activity against *T. cruzi* is comparable with that of the highly active POSA and ketoconazole.^141^ However, its extremely short terminal half-life precluded studies of in vivo trypanocidal assays in murine models. In an acute murine model using Y strain–infected mice, treatment with free albaconazole showed lower efficacy than treatments with POSA, ketoconazole, and RAVU because no cure was observed. However, survival was similar to that with ketoconazole and RAVU.^142^ Interestingly, Y strain–infected mice treated with albaconazole in nanocapsules (120 mg/kg/day administered subcutaneously) had 100% survival and 60% negativation during a period > 120 days, although no parasitological cure was observed.^142^ In a dog model, which is more appropriate for albaconazole assays,^143^ the compound resulted in cure in 100% of animals inoculated with *T. cruzi* strain Y when they were treated for long periods (90 days). Unfortunately, although it was very effective in suppressing parasite proliferation in animals infected with the Berenice-78 *T. cruzi* strain, no parasitological cure was observed (150 days of treatment). Albaconazole is a good candidate for the treatment of human CD because of its remarkably long half-life in humans and the ability for long-term treatment (60–150 days) with minimal toxicity.

##### Voriconazole.

Voriconazole, an antifungal triazole derivative, has demonstrated in vitro and in vivo activity in a murine model of acute *T. cruzi* infection,^144^ significantly reducing the peak of parasitemia, increasing lifespan, and decreasing mortality compared with nontreated mice. Unfortunately, treatment with voriconazole proved significantly less effective than the reference drug BZ in parasitemia reduction. However, the use of voriconazole for CD treatment is still possible because only insignificant adverse events have been reported, and its low toxicity profile allows potentially higher doses to overcome the relatively low potency.

##### Fenarimol analogues.

Fenarimol, a nontoxic plant fungicide, has potent activity against *T. cruzi* in whole-cell in vitro assays. Lead compounds suppressed blood parasitemia to virtually undetectable levels after one daily oral dose in murine models of *T. cruzi* infection.^145^ An immunosuppressive model of subchronic *T. cruzi* infection showed that the efficacy of two fenarimol analogues was comparable with POSA and better than BZ.^146^ A lead optimization consortium headed by DNDi aimed to characterize two preclinical candidates from the fenarimol series showing curative efficacy in murine models of CD.^147^ The project was in its nonregulatory preclinical phase, with further profiling of candidates necessary before nominating one candidate for further regulatory preclinical development. The objective was to perform good laboratory practice safety studies and chemical, manufacturing, and control studies with the selected candidate compound to file a formal investigational new drug application and to move the candidate to first-in-man studies. Unfortunately, this project was stopped because of the lack of sustained efficacy with azoles (E1224 and POSA) in clinical trials for CD.

#### Oxaborole derivates.

Anacor Pharmaceuticals (Palo Alto, CA) has collaborated with DNDi, Murdoch University, and Epichem (Perth, Australia) to identify a new class of oxaborole compounds for CD treatment. The in vitro screening of *T. cruzi* against the boron-containing compound collection, provided by Anacor Pharmaceuticals, identified a number of compounds more potent than BZ,^148^ and a recent study demonstrated that Oxaborole SCYX-7158 cured 100% of mice infected with the susceptible strain Brazil when administered for 40 consecutive days.^85^ The phase I clinical study with SCYX-7158 for treatment of human African trypanosomiasis was completed in 2015, and and the phase II/III trial started in 2016.^149^

#### Inhibitors of polyphosphate metabolism (Bisphosphonates).

Bisphosphonates are used to prevent bone resorption in humans. Risedronate, a potent bisphosphonate, has in vitro^150^ and in vivo activity against *T. cruzi*.^151^ Bisphosphonates inhibit the enzyme farnesyl pyrophosphate synthase of *T. cruzi*.^150^ A murine model of acute CD using the Y strain demonstrated that treatment with risedronathe reduced parasitemia and increased survival.^151^ Another murine study investigated the role of this drug in the development of chronic chagasic cardiomyopathy using the nitrofuran/nitroimidazole-susceptible strains of *T. cruzi* Brazil and Tulahuen^152^ and found a significant reduction in the mortality of mice infected with the Brazil strain but no effect on the survival of mice infected with the Tulahuen strain. Unfortunately, no cure was reported in these studies.

#### Purine synthesis inhibitors (Allopurinol).

Allopurinol (4-hydroxypyrazolo[3,4-d]pyrimidine) is used for treatment of hyperuricemia in humans. The in vitro^153^ and in vivo^154^ anti-*T. cruzi* activities of allopurinol were described three decades ago. In a murine study, the compound induced highly significant reductions in parasitemia and mortality rates and increased survival time. In humans, the data regarding allopurinol efficacy are conflicting. One study involving patients with chronic CD demonstrated that oral allopurinol was as effective as treatment with nitrofurans, without the side effects.^27^ However, other studies found that allopurinol was ineffective in patients with acute^155^ or chronic CD.^74,75^ The differences in *T. cruzi* strain susceptibility to drugs could explain the discrepancy and still need to be evaluated.

#### Amidine compounds and analogues.

Recent research at Fundação Oswaldo Cruz, Brazil, addressed the effects of several amidine analogues against *T. cruzi*. In vitro and in vivo activities were found for arylimidamides^156–160^ and diamidines using experimental murine models of acute *T. cruzi* infection.^161,162^ The most potent arylimidamide, DB766, exhibits strong trypanocidal activity and excellent selectivity for bloodstream trypomastigotes and intracellular amastigotes (Y strain).^159^ DB766 also exerts striking effects on strains susceptible and naturally resistant to BZ and displays higher activity in vitro than the reference drugs. In in vivo assays, DB766 effectively reduces the blood and cardiac tissue parasite load and has similar efficacy to BZ in murine models of *T. cruzi* infection employing the Y (partially resistant) and Colombiana (resistant) strains. DB766 ameliorates ECG alterations, reduces hepatic and heart lesions induced by *T. cruzi*, and provides 90–100% protection against mortality, similar to BZ.

#### Inhibitors of the kinetoplastid proteasome.

Recently, a selective inhibitor of the kinetoplastid proteasome (GNF6702) with unprecedented in vivo efficacy against CD, leishmaniasis, and sleeping sickness was described.^163^ This compound cured mice in all three models of infection through a noncompetitive mechanism. It is well-tolerated in mice, has good pharmacokinetic properties, and does not present activity in panels of human receptor, enzyme, and ion channel assays. Importantly, the dose used in experimental chronic CD was significantly smaller than the dose of standard drug (twice-daily at 10 mg/kg to inhibitor versus 100 mg/kg once-daily to BZ). This inhibitor is currently being evaluated in preclinical toxicity studies. However, because the CL susceptible strain was used, it is very important to analyze the activity of GNF6702 inhibitor against *T. cruzi* strains resistant to BZ and NFX.^164^

### Benznidazole encapsulation in liposomes, nanoparticles, and other microparticles.

A promising way to increase the activity and/or selectivity of drugs is to microencapsulate them in biodegradable polymers that continuously release their content over time. The few studies that have evaluated BZ encapsulated in delivery systems, especially in liposome vesicles,^165–168^ attempted to improve BZ pharmacokinetics to decrease the therapeutic dose and diminish the side effects. Another strategy to increase activity/solubility is to encapsulate BZ in microparticles such as chitosan.^169^ The new formulations, such as liposomes, nanoparticles, and other microparticles, are expected to improve the pharmacokinetics and pharmacodynamics of actual CD therapies by decreasing the total dose of drug used, minimizing the toxicity profile, reducing the appearance of resistance, and increasing the tissue concentration. The main limitation of the use of such technologies is that the encapsulation burden it might not be sufficient to give a dosage fully efficient.

The first study using a multilamellar liposomal formulation for BZ was performed at the outset of the 2000s.^165^ Kupffer cells in the liver and spleen macrophages are major natural targets for multilamellar liposomes. In theory, BZ encapsulated in multi-lamellar liposomes should increase the amount of drug delivered to infected Kupffer cells, and hence, eradicate the amastigote nests in the cytoplasm of Kupffer cells.^167^ To test this hypothesis, free BZ or liposomal BZ formulations (0.2 mg/kg BZ) were injected intramuscularly, subcutaneously, or intravenously in mice. Unexpectedly, the increased liver uptake of BZ had no effect on parasitemia levels; therefore, the relationship between increased selectivity for an infected tissue and therapeutic effect is not always straightforward. The main obstacles for BZ via a delivery system for CD treatment are the disseminated localization of *T. cruzi*,^170^ low hydrosolubility of BZ, and absence of weak acid-base behavior, which hinders BZ retention in phospholipid bilayers on dilution and the use of an active loading method to obtain a high drug/lipid ratio.^165^ However, the development of parenteral BZ formulations that are more water soluble may help to overcome these limitations.^171,172^

The ongoing BERENICE project, a European-sponsored and -funded project comprising eight public and private institutions in Spain, Portugal, France, Brazil, and Argentina aims to obtain a more effective, better-tolerated, and cheaper BZ formulation to cure CD using nanotechnology (http://www.berenice-project.eu/). The first approach is to develop novel BZ lipid-based drug delivery systems; these include solid lipid nanoparticles, which are particulate drug carrier systems able to achieve a sustained release of the drug, thus minimizing its adverse effects, and small unilamellar vesicles, which are tailored according to size, morphology, supramolecular structure, and response to external stimuli, to improve the pharmacological properties of the active pharmaceutical ingredient. The second approach is to develop a new galenic form of BZ for sublingual delivery to allow the drug to reach the bloodstream directly, avoid hepatic first-pass effects, and obtain an optimum concentration. The third objective is to establish a clinical trial platform to evaluate the tripanocidal efficacy of the new nanoencapsulated BZ alone or in combination with new ergosterol inhibitor candidates.

### Pharmacokinetic and biodistribution of benznidazole.

BZ is administered orally (two or three daily doses) and is rapidly absorbed from the gastrointestinal tract. On average, peak plasma levels (2.5 μg/mL) are reached 3–4 hours after drug administration in humans, and the average relative BZ bioavailability is 91.7%.^173–176^ In dogs, peak plasma concentration was attained rapidly (1–5 hours), with complete bioavailability. Similar bioavailability was also observed in mice, and peak concentration was usually achieved by 30 minutes.^177^ NFX presents a similar profile because after 1–3 hours after oral adminstration of 15 mg/kg to man the peak plasma levels of 2–3 μg/mL was found; in dogs, the maximum plasma concentration 4.3 μg/mL is attained within the first 2 hours and is similar to that found in rats.^178^

The BZ is readily metabolized by hepatic cytochrome P450 reductase to generate toxic products, and the half-life of BZ is 12 hours in humans.^173,175,176^ The NFX is biotransformed via a presystemic, first-pass effect, generating several unidentified metabolites, and its half-life is 3 hours.^175^ In animal models, the half-life of BZ is as follows: 90 minutes in mice, 4–5 hours in sheep, and 9–11 hours in large crossbred dogs. The pharmacokinetics of BZ in mice, sheep, and dogs showed considerable binding of BZ to plasma proteins, at 39% in mice, 59% in dogs, and 42% in sheep,^177^ compared with 58%^179^ and 44%^173^ in human plasma. Since only 5% of administered BZ is recovered unchanged in the urine, most of the drug is eliminated through metabolic products, although other mechanisms (e.g., biliary and fecal excretion) cannot be excluded. It is likely that ring cleavage also occurs.^177^ To the best of our knowledge, the more complete study about BZ biodistribution was performed in mice by Perin et al.,^180^ which BZ concentration ranged from 0.1 to 100.0 μg/mL for plasma, spleen, brain, colon, heart, lung, and kidney and from 0.2 to 100.0 μg/mL for liver after oral administration of BZ. There were similar times to maximum concentration in organs, with means of 40 minutes. Pharmacokinetics studies of NFX, showed that after oral administration this drug labeled with radioactive sulphur-^35^S to rats, the drug is almost completely metabolised.^181^ This finding is corrobored by a study that demonstrated that only 0.5% of NFX is excreted in the urine after oral administration.^178^

Despite the widespread use of BZ, important data regarding pharmacokinetics and pharmacodynamics are still lacking. New proposals to bridge these gaps will be fulfilled in the future. A pharmacokinetics study in adult patients was completed in May 2015 (ClinicalTrials.gov Identifier: NCT01755403).^182^ This clinical trial show that in the standard regimen of 5 mg/kg/day of BZ divided into two doses (2.5 mg/kg/12 hour), only 5.4% of the observed BZ trough concentrations were below 3 mg/L; 20% of them were within the optimal range (3–6 mg/L), but most of them (74.54%) were above 6 mg/L. Furthermore, results from simulations showed that the usual dose regimen of 2.5 mg/kg/12 hour would allow achievement of the target of 3 mg/L during the whole interdose interval in almost all of the treated subjects. In addition, a phase I, open-label, nonrandomized pharmacokinetic study of BZ using eight healthy adult volunteers, performed by the BERENICE project, show similar results.^183^ These findings support the rationale of proposing a lower BZ dose.

## CONCLUSION

Because no safe and fully effective drug is available for CD treatment, and few drugs are being evaluated in clinical trials, research to find new treatments for CD is urgently needed. Drug development is expensive and time consuming; therefore, until few years ago “Big Pharmas” had little interest in the development of new drugs for CD, a highly neglected tropical disease. Research for new anti-*T. cruzi* drugs relies mainly on existing drugs for other diseases (label extension and piggyback discovery strategies). An alternative approach is modification of the current chemotherapy drugs for CD to diminish their toxicity and/or increase their trypanocidal efficacy. Another alternative is improving the selectivity of current drugs for CD chemotherapy, as proposed by the BERENICE project. Fortunately, there has been increasing interest in research for new CD drugs, as evidenced by ongoing or recently completed clinical trials^184^ and the recent programs for development of new chemical entities for the treatment of Tropical Neglected Diseases launched by companies such as Novartis,^185^ GSK,^186^ Pfizer (Anacor),^187^ Jhonson & Jhonson (Janssen),^188^ Sanofi,^189^ and Merck & Co., Inc.^190^

It is also possible to decrease the total BZ or NFX dose to reduce their toxicity and the subsequent effect on treatment interruption. Bustamante et al.,^85^ using a combined, reduced dosing treatment regimen and intermittent protocol observed 100% cure of mice infected with *T. cruzi*. In addition, this finding enables the possibility of improve the effectiveness of BZ and NFX through the increase of time treatment.

POSA and RAVU, the two more promising drugs for CD treatment recently evaluated in clinical trials, had disappointing results in phase II clinical trials. However, they remain promising because they have low toxicity in humans and are approved for use in humans, which can reduce the time and investment for drug development. They could also be combined with the drugs currently used for CD, i.e., BZ and NFX, and with other trypanocidal drugs, mainly those with reported synergic activity in in vitro and in vivo studies.

### Transparency declaration.

This study was supported by the European Comission under the Health Innovation Work Program of the 7th Framework Program and by CAPES/Brasil, Programa Ciência Sem Fronteiras and Professor Visitante Nacional Senior. The funders had no role in study design, data collection and analysis, decision to publish, or preparation of the manuscript. The authors have no conflict of interests.
